# Putting the Goal Back into Grit: Academic Goal Commitment, Grit, and Academic Achievement

**DOI:** 10.1007/s10964-020-01348-1

**Published:** 2020-11-17

**Authors:** Xin Tang, Ming-Te Wang, Filomena Parada, Katariina Salmela-Aro

**Affiliations:** 1 Faculty of Educational Sciences, University of Helsinki, Helsinki, Finland; 2 School of Education, Department of Psychology, and Learning Research and Development Center, University of Pittsburgh, Pittsburgh, PA USA

**Keywords:** Grit, Goal commitment, Achievement, Goal, Perseverance

## Abstract

Grit has recently been challenged for its weak predictive power and the incompleteness of its measurement. This study addressed these issues by taking a developmental, person-oriented approach to study academic-related goal commitment and grit and their effects on academic achievement. Using longitudinal data among Finnish eighth and ninth graders (*n* = 549, 59.4% female, age = 14–16), the longitudinal changes in grit and academic goal commitment profiles were investigated through latent profile and latent transition analyses. Four profiles were identified across two grades: High committed-persistent and moderate consistency (~17%), Moderate (~60%), Low committed-persistent and moderate-low consistency (~8%) and Extremely low committed-persistent and moderate-low consistency (~12%). The students in the High committed-persistent and moderate consistency profile had the highest academic achievement of all the profiles when controlled for gender, socioeconomic status, conscientiousness, and academic persistence. The results revealed that students’ profiles changed between the eighth and ninth grades, with more than one-third of the High committed-persistent and moderate consistency adolescents dropping from this group. Further analysis showed that the profiles varied by educational aspiration, gender, and socioeconomic status. These findings imply that the combination of grit and academic goal commitment influences academic achievement; however, this combination is less common, unstable, and affected by internal and external factors. The study provided important implications on the weak grit effect and the ways to improve it.

## Introduction

Defined as the trait of passion and perseverance for long-term goals, the concept of grit has received a great deal of attention and examination in the academic literature due to its role in performance (Duckworth et al. 2007). However, there has been increasing criticism of its construct and predictive validity. For instance, grit only shares weak association with achievement, a relation that weakens when accounting for conscientiousness or self-control (Credé 2018; Credé et al. 2017). Moreover, scholars have noted issues regarding the alignment between the concept of grit and how it is measured (Credé 2018). For instance, although goals have been defined as a central component of grit, grit scales have rarely addressed them. Finally, the developmental characteristics of samples or goals may add complexity to identify grit effect; yet, extant research has largely relied on cross-sectional data. In response, this study aimed to bring the goal—the neglected component of grit—back into research by using a person-oriented approach to examining goal commitment and grit, and their associations with achievement. The present study also aimed to examine the antecedents, stability, and transition of grit-goal commitment profiles longitudinally to understand their immediate and long-term roles in student achievement.

### Grit and Achievement

Grit was reported to associate with a wide range of achievement indicators across different domains in earlier studies (Duckworth et al. 2007; Eskreis-Winkler et al. 2014); nevertheless, recent meta-analyses have indicated that grit is only weakly associated with achievement when evidence is aggregated (Credé et al. 2017; Lam and Zhou 2019). The unique role of grit has also been questioned, as research has shown that grit—more specifically, grit’s perseverance facet—is highly correlated with conscientiousness (Rimfeld et al. 2016; Schmidt et al. 2018). However, some recent findings still have shown promising signs of grit’s predictive relation with achievement. For example, two longitudinal studies in Finland (grades 6–9; Tang et al. 2019) and China (grades 4–6; Jiang et al. 2019) found that grit, especially its perseverance facet, predicted adolescents’ academic achievement after controlling for previous achievement, conscientiousness/self-control, and other demographics. Another nationally representative employed-sample study in Germany (Lechner et al. 2019) also reported an effect of grit on career success (indexed by income, job prestige, and job satisfaction) and career engagement after accounting for cognitive ability and sociodemographic variables. Moreover, a newly published study espouse that grit (as compared to cognitive and physical ability) was the strongest predictor of completion of a highly-intensive training program and graduation status among cadets at West Point Academy (Duckworth et al. 2019). These findings suggest that the context (e.g., culture, sample age, physical setting) must be considered when examining the associations between grit and achievement.

Importantly, some studies have shown that the grit-achievement association could be enhanced in some circumstances, thereby positioning the concept as a potential point of intervention. One study found that the grit effect was more significant at the two ends of the cognitive ability continuum in a nationally representative adult sample (Light and Nencka 2019). For adults with low cognitive ability, those with high levels of grit had high education attainments; however, this same effect was not found among adults with average cognitive ability. Other researchers have found that when the grit scale is modified to be domain specific, the association between achievement and the domain-specific grit scale is higher than the association found for the original measure (Clark and Malecki 2019; Cormier et al. 2019; Schmidt et al. 2019). As such, future studies of grit are warranted to further investigate in which conditions and to what extent grit provides incremental validity for achievement (Credé 2018).

### Goal, Commitment, and Grit

To find strong grit effects, the present study investigates a neglected component of grit— the *goal*. Although goals have been used to define grit, the term “goal” and the role of goals have been largely ignored in the measurement and discussion of grit. For instance, the most frequently used measure of grit (Duckworth and Quinn 2009) includes only one goal-related item (i.e., “I often set a goal but later choose to pursue a different one.”). This lack of goal-related items not only raises concerns about conceptualization and measurement (Credé 2018), but it also hinders the ability of researchers to identify the strength of grit effects (Jordan et al. 2019). According to goal-setting theory (Locke and Latham 2002), successful goal pursuit requires two effective elements: goal setting and goal implementation (Kruglanski et al. 2015; Oettingen and Gollwitzer 2009). Goal setting is the process by which a goal is constructed, selected, and committed to (Locke and Latham 2002) and goal implementation is the action phase of goal pursuit in which an individual strives to achieve their chosen goal (Oettingen and Gollwitzer 2009). In other words, goal setting characterizes the direction of goal pursuit, whereas goal implementation reflects the energizing part of goal pursuit. To date, though, empirical and theoretical literature addressing grit has mostly explored goal implementation while largely ignoring goal-setting processes.

To find a strong association between grit and achievement in a certain domain, one needs to ensure that students hold achievement in that specific domain as a primary goal. For example, if achieving a good grade on a school math test is not a goal for a student, grit will not contribute to their academic achievement in math, no matter how much overall grit this student may have. Recent re-conceptualizations of grit have identified this contextualized goal-grit-achievement issue as the main reason behind the low associations between grit and achievement. According to Jordan et al. (2019), grit should be regarded as the context-specific ability to (a) set and pursue long-term goals and (b) readjust short-term goals and goal attainment strategies in the face of challenges and difficulties. Thus, without information on goals and their contents, grit is an undirected energy that is easily drained (Jordan et al. 2019). Consequently, it is not a surprise that many researchers have found only weak associations between grit and achievement.

Returning to the goal setting process of grit, adolescents and adults typically have multiple long-term goals across several domains (Salmela-Aro et al. 2007). However, goal pursuit requires energy and resources; hence, people have to prioritize their commitment to the goal in order to achieve it (Klein et al. 2013; Kruglanski et al. 2015). To achieve success or excellence, people tend to focus on and devote much of their time, energy, and resources to domain-specific goals (Kruglanski et al. 2002; Salmela-Aro 2009). In other words, people may approach multiple long-term goals at the same time, but they likely do not commit to these goals equally. Goal commitment has been defined as a volitional psychological bond reflecting dedication to a specific goal (Klein et al. 2013). According to Locke and Latham (2002), the goal–performance relationship is strongest when people are highly committed to their goals. Therefore, even if an individual is regarded as gritty, their grit cannot contribute to their academic achievement if academic achievement is not the goal, or their commitment to this goal is weak.

This study focuses on the relation between commitment of academic-related goals (i.e., concrete goals related to academic learning and achievement) and academic achievement. Preliminary evidence supports the assumption that the combination of academic-related goal commitment and grit will result in strong academic achievement. For example, one study found that a sample of student athletes had a high level of sport grit, but low levels of school grit and general grit (Cormier et al. 2019). In terms of student academic achievement, it was found that school grit—not sport or general grit—was the contributing factor (Cormier et al. 2019). Thus, this study intends to supply academic-related goal commitment into grit and aims to see they have a stronger effect on academic achievement.

### The Change of Goal Commitment-Grit Compound and the Underlying Reasons

Although it is important to understand the combination of goal commitment and grit, it is also essential to examine dynamic changes in this relation over time. The formation of goals is largely affected by contextual factors such as age or educational level (Salmela-Aro 2009). As people move through life, they go through different developmental stages that often require them to explore, select, and revisit their goals (Salmela-Aro 2009). Adolescents and young adults explore their identity and are in the process of constructing their long-term goals (Hofer 2010; Salmela-Aro 2009). Amidst this process, the secondary school years (i.e., grades 7–12; ages 13–18) form a critical period for adolescents in terms of developing goals and grit. Research has found that leisure goals are most desired in early adolescence (age 10–14), while education-related goals increase during mid-adolescence (age 15–17) and then decline in late adolescence (Massey et al. 2008). Over the years, adolescents also become more realistic about their goals and abilities to attain them (Massey et al. 2008). To date, the development of grit has remained understudied. Several cross-sectional studies have suggested that grit increases with age (Credé et al. 2017) and a few exclusive longitudinal studies have indicated that grit may be less stable among early adolescents (grades 4–6; test–retest *r* = 0.40; Jiang et al. 2019) but moderately stable among mid-adolescents (grades 8–9; *r*s~0.60; Duckworth and Quinn 2009; Park et al. 2020). However, given the research aims of this study, how grit and goal commitment develop as a whole during the secondary school years is largely unknown.

As such, this study aims to identify how grit and goal commitment develop during the secondary school years using a Finnish sample of eighth- and ninth-grade students. As noted above, adolescents in their lower secondary years (grades 7–9) experience changes in personal goal-setting (Massey et al. 2008). In the Finnish education system, these changes coincide with the ninth grade, marking the end of compulsory education (OECD 2016). After this period, Finnish adolescents enter academic high school, vocational high school, or work life (Tuominen-Soini et al. 2012). This makes the eighth and ninth grades critical pre-transition years during which Finnish adolescents form and revisit their goals for the future. In other words, the ninth grade marks one of the first consequential crossroads regarding future career development for Finnish adolescents (Vasalampi et al. 2010).

Moreover, this study uses a person-oriented approach to examine the combination of academic goal commitment and grit longitudinally. Whereas variable-oriented approaches focus on aggregated sample characteristics or the relationships between variables, person-oriented approaches focus more on individuals. The person-oriented approach aims to identify subgroups of individuals so as to study them together as an undivided whole (Bergman and Trost 2006). Following the guidance of extant research, the present study examined two facets of grit independently (i.e., perseverance of effort and consistency of interest; Credé 2018; Guo et al. 2019). In comparison to the variable-oriented approach, the person-oriented approach better reflects the natural configuration of the combinations of variables, particularly for multiple variables (Bergman and Trost 2006); thus, a person-oriented approach can illuminate how goal commitment, grit-perseverance, and grit-consistency function together as well as how these combinations are associated with achievement. In addition, recent developments of latent transition analysis have enabled further examination of the changes, stability, or transitions among these combinations (Lanza et al. 2003). Consequently, the person-oriented approach as a whole provides not only proportional information on variable combinations, but also stability and transition information on these combinations. This approach can also test and ensure the equivalency of measures, constructs, and the interpretation and distributions of combinations across the years, which are particularly important for longitudinal studies (Morin and Litalien 2019).

In addition to identifying and monitoring changes in the profiles of academic-related goal commitment and grit over time, this study was also interested in understanding which factors influence the formation of these profiles. This line of inquiry may help researchers further understand the mechanism of change in goal commitment and grit combinations. This study focused on the role of educational aspirations (i.e., the highest degree one aims to obtain), gender, and socioeconomic status (SES) given their general impact on goals, personality, and achievements (Flunger et al. 2016; Parker et al. 2016). Previous studies have shown that adolescents who are girls, have high educational aspirations, or come from a high SES family tend to be highly committed to educational goals (for review, see Massey et al. 2008). In general, researchers have not identified significant gender differences grit (Credé et al. 2017; Duckworth and Quinn 2009); however, educational aspirations (e.g., Verdesco 2016) and SES (e.g., Usher et al. 2019) tend to both share a positive association with grit. Moreover, the present study included conscientiousness and academic persistence as covariates to examine the unique role of grit. Conscientiousness—that is, the personality trait of being thorough, industrious, and self-controlled—has been associated with grit (Duckworth et al. 2007), and academic persistence refers to one’s motivational tendency when facing difficult school tasks (Tuominen-Soini et al. 2012). Both of these factors are conceptual and instrumental closed constructs of grit, and they are included in this study so as to isolate the exclusive contributions of grit.

## Current Study

By noting that the combination of goal commitment and grit would enhance grit’s effects on achievement, this study examines the combined effects of goal commitment and grit on achievement. Using a person-oriented approach, this study created profiles of academic goal commitment and grit and then investigated their differential associations with academic achievement. To understand the mechanisms and contextual reasons behind these combinations, the current study also explored shifts in goal-grit profiles longitudinally. In sum, the study addressed four research questions:

First, what profiles of grit and academic goal commitment can be identified? The study expected some students to have high levels of academic goal commitment and grit, some students to have a low level of academic goal commitment but a high level of grit, and some students to have low levels of both academic goal commitment and grit.

Second, how do the profiles change between the eighth and ninth grade? Given that adolescents are developmentally prone to changing or honing their goals, students shifting between these goal-grit profiles over time were expected (Massey et al. 2008).

Third, how do these profiles differ in terms of academic achievement? Of these profiles, this study expected that students with high levels of academic goal commitment and grit will have the highest academic achievement (Tang et al. 2019).

Fourth, do educational aspirations and demographic variables (e.g., gender and socioeconomic status) affect the formation of the profiles? The current study expected that students with high educational aspirations and high SES will be more likely to be in the profile characterized by high grit and academic goal commitment. It also expected to find no gender differences between these profiles (Duckworth and Quinn 2009).

## Methods

The sample in the present study consisted of eighth and ninth graders (age = 14–16) who participated in a large Finnish longitudinal study (from the sixth grade to the ninth grade). This study used two waves of data (i.e., eighth and ninth grade), with grit being measured from the eighth grade. In total, 549 students (59.4% girls, 2.7% no report of gender) who reported grit and goal commitment in the two waves of the study were included in the analysis, as the current study focused on both cross-sectional and longitudinal latent profiles.

Participation in the project was voluntary, and informed consent forms were collected from both the students and their parents. The study questionnaire was administered during school hours and took about an hour to complete. Data on grit, academic goal commitment, GPA, SES, gender, conscientiousness, and academic persistence were collected in both waves.

### Measures

#### Grit

The students’ grit was measured using the short version of the Grit scale (eight items; Duckworth and Quinn 2009) in the eighth and ninth grades. This scale has four perseverance of effort items (PE: e.g., “I am diligent”) and four consistency of interest items (CI: e.g., “I often set a goal but later choose to pursue a different one”). The items were measured on a Likert scale of 1 = *not at all like me*, to 5 = *very much like me*. The Cronbach alpha of this scale was acceptable (for the eighth grade: α_CI_ = 0.70, α_PE_ = 0.78; for the ninth grade: α_CI_ = 0.72, α_PE_ = 0.79).

#### Academic-related goal commitment

Academic-related goal commitment was measured using the revised version of the Personal Project Analysis Inventory (Salmela-Aro and Nurmi 1997). Students were first asked to write down one academic goal related to school, education, or academic achievement (e.g., “I want to keep my grade average at 9.5^1^”, “I want to be admitted to a good high school”). Then, students were asked to appraise their commitment to the goal they had identified (see Flunger et al. 2016). Goal commitment was measured using three items (e.g., “How committed are you to this goal?”, “How important is this goal?”, “I really believe that this is an important goal”; α = 0.84 and α = 0.85, respectively for the eighth and ninth grades). The items were rated on a seven-point Likert-type scale, ranging from 1 = *not at all* to 7 = *very much*.

#### Educational aspirations

The students’ educational aspirations were measured by asking them to indicate the highest degree they expected to attain. Five choices were given: university, polytechnic, academic upper secondary school, vocational upper secondary school, or other. In this study, this variable was recoded as 1—vocational upper secondary school, 2—academic upper secondary school, 3—polytechnic, 4—university, whereas the rest were coded as missing.

#### Academic achievement

The students’ grades were obtained from school records on each subject in each term of the eighth and ninth grades. After this, a yearly GPA was calculated on the basis of the two terms’ records.

#### Covariates

Gender, SES, conscientiousness, and academic persistence were assessed using self-report items. SES was determined by asking participants to rate their family’s financial situation (1 = *bad* to 5 = *good*). Conscientiousness was measured using two items (“I can be careless sometimes” and “I tend to be lazy”; α = 0.64 and α = 0.62, respectively for the eighth and ninth grades) on a five-point Likert-type scale (i.e., 1 = *Completely disagree* to 5 = *Completely agree*), and these responses were reverse-coded for further analyzes. Academic persistence was adapted from the academic withdrawal dimension in the Achievement Goal Orientation Scale (Niemivirta 2002). Three items (e.g., “I have realized that I give up easily if school tasks are difficult.”; α = 0.81 and α = 0.82, respectively for the eighth and ninth grades) were rated on a seven-point Likert scale (1 = *Not true at all* to 7 = *Very true*). These responses were also reverse coded for further analyzes.

### Analysis Strategy

Latent profile analysis (LPA) and latent transition analysis (LTA) were performed to estimate the profiles of grit and goal commitment across two grades and examine transitions between these profiles. Before running the LPA and LTA, factor scores (estimated in standardized units as M = 0, SD = 1) of grit and academic goal commitment were saved using a longitudinal measurement invariance model (see Supplementary Table S1). The use of the factor score, as opposed to the scale score, enables partial control over measurement errors while simultaneously retaining the underlying nature of the measurement model. All data^2^, syntax and outputs can be found at https://osf.io/3nz4e/. In this study, all the models were estimated using Mplus 8.2 (Muthén and Muthén 2018).

#### Latent profile analyses

To decide on the numbers of profiles to be used in the present study, the LPA models for each wave’s data were first estimated separately to ensure that the same number of profiles could be retained at each time point. As three indicators were used (i.e., academic goal commitment, grit-PE, grit-CI), five LPA solutions were freely estimated using the means and variances of the indicators. Determining the optimal number of profiles required both theoretical meaningfulness and statistical adequacy. For each profile solution, the following criteria were evaluated: Akaike’s information criterion (AIC), the Consistent AIC (CAIC), the Bayesian information criterion (BIC), and the adjusted Bayesian information criterion (ABIC). The Vuong–Lo-Mendel–Rubin test *(*VLMR*)* and the Lo-Mendell–Rubin adjusted LRT test (LMR) were used to compare the k–class model with k-1–class model. Lower AIC, CAIC, BIC and ABIC values indicate a better fit, and significant (*p* < 0.05) test results indicate a higher number of profiles. In addition, “elbow plots” were used to illustrate the changes in these four information criteria in order to facilitate the decision (e.g., Gillet et al. 2017). The point after which the slope flattens typically suggests the optimal number of profiles. Finally, the entropy values were also considered to indicate classification quality. Entropy values range from 0 to 1, with 0 corresponding to randomness and 1 to perfect classification.

#### Latent transition analyses

After the selection of the optimal number of profiles in each grade, the two LPA solutions were integrated into a longitudinal LPA model to be tested for profile similarity. Following the earlier guidance (Morin and Litalien 2017), *configural similarity* was first examined to determine whether the same number of profiles could be identified at each time point. Then the *structural similarity* of the longitudinal LPA model was examined by constraining the means of the profile indicators to remain equal across the two time points. The *dispersion similarity* of the profiles was then investigated if the prior similarity test was verified. This third similarity test (i.e., dispersion similarity) imposed equality constraints on the variances of the profile indicators across the time points. Lastly, the *distributional similarity* of the profiles was examined by constraining the class probabilities of being equal across time points if the third one held. Each of these models’ fits were compared using the CAIC, BIC, and ABIC. It has been suggested that at least two out of three indices be lower for the more ‘similar’ model to support the profile similarity hypothesis (Gillet et al. 2017). Once the most similar model was chosen, it was converted into a longitudinal LTA model (Morin and Litalien 2017; Nylund-Gibson et al. 2014) to examine the profile membership’s stability and transitions.

This final longitudinal LTA model enabled us to add covariates, predictors (e.g., educational aspiration) or outcomes to determine whether the associations between the profiles and their predictors (“predictive similarity”) and outcomes (“explanatory similarity”) remained the same across time points (Gillet et al. 2017; Morin and Litalien 2017). Following the earlier suggestions (Asparouhov and Muthén 2014), the manual auxiliary three-step approach was used in this testing.

### Missing Data Handling

The study originally had complete data on 1174 students in the eighth grade and 857 students 1 year later in the ninth grade. The missing completely at random test (MCAR; Little 1988) revealed that data were not missing completely at random. The participants who stayed at both eighth and ninth grades (*N* = 610; 549 of them had common data on grit and goal commitment) and those who stayed at the eighth grade only (*N* = 564), did not differ in terms of grit-consistency (*t* = −0.39, *p* = 0.69), SES (*t* = 1.44, *p* = 0.15), academic persistence (*t* = 1.77, *p* = 0.07) or conscientiousness (*t* = 0.15, *p* = 0.88) in the eighth grade but those who continued to the ninth grade had higher grit-perseverance (*t* = 2.12, *p* < 0.05), academic goal commitment (*t* = 4.45, *p* < 0.001), and educational aspiration (*t* = 3.70, *p* < 0.001), and GPA (*t* = 5.31, *p* < 0.001) in the eighth grade, and they were also more likely to be girls rather than boys (*χ*² = 2.39, *p* < 0.05). To handle the missing data, the robust maximum likelihood estimator (MLR) was used throughout the analyzes.

## Results

The zero-order correlations among the studied variables are shown in Table 1.

**Table 1:** Correlations between variables used in present study

Variable	1	2	3	4	5	6	7	8	9	10	11	12	13	14	15	16
1. Gender	–															
2. Academic goal commitment (T1)	−0.11*	–														
3. Consistency of Interest (T1)	0.03	0.14*	–													
4. Perseverance of Effort (T1)	−0.07	0.45**	0.20**	–												
5. Social economic status (T1)	0.11*	0.21**	0.08	0.24**	–											
6. Conscientiousness (T1)	0.08	0.06	0.50**	0.44**	0.16**	–										
7. Academic Persistence (T1)	0.08	0.12*	0.55**	0.38**	0.12*	0.47**	–									
8. Academic Achievement (T1)	−0.18**	0.23**	0.09	0.33**	0.10*	0.19**	0.36**	–								
9. Education aspiration (T1)	−0.06	0.28**	−0.01	0.21**	0.09	0.06	0.14**	0.44**	–							
10. Academic goal commitment (T2)	−0.19**	0.30**	0.04	0.29**	0.08	0.02	0.07	0.13*	0.18**	–						
11. Consistency of Interest (T2)	0.05	0.11*	0.54**	0.27**	0.13*	0.40**	0.32**	0.06	0.01	−0.08	–					
12. Perseverance of Effort (T2)	−0.09	0.32**	0.25**	0.55**	0.10*	0.32**	0.27**	0.23**	0.16**	0.42**	0.09	–				
13. Social economic status (T2)	0.09*	0.14**	0.10*	0.10*	0.64**	0.14**	0.15**	0.05	0.12*	0.11*	0.04	0.12**	–			
14. Conscientiousness (T2)	0.04	0.00	0.43**	0.31**	0.06	0.53**	0.36**	0.14**	0.03	−0.20**	0.67**	0.31**	0.07	–		
15. Academic Persistence (T2)	0.11*	0.19**	0.40**	0.33**	0.14**	0.43**	0.67**	0.32**	0.19**	0.16**	0.46**	0.36**	0.09*	0.39**	–	
16. Academic Achievement (T2)	−0.15**	0.24**	0.06	0.34**	0.13**	0.19**	0.33**	0.92**	0.49**	0.21**	0.08	0.29**	0.07	0.15**	0.36**	–
17. Education aspiration (T2)	−0.12*	0.27**	−0.10	0.17**	0.16**	−0.04	0.12*	0.43**	0.65**	0.22**	0.01	0.10*	0.14**	−0.01	0.10	0.48**

T1: 8th grade; T2: 9th grade

*p < 0.05; **p < 0.01

### Latent Profiles of Goal Commitment and Grit

Fit indices and theoretical meaningfulness were used to determine the optimal number of profiles. In both grades, all fit indices continued improving as profiles were added (see Table S2). However, the improvement of fit indices became small after the four-profile solution (especially for ninth grade data; see Figure S1 & S2). A substantial increase in entropy values was also observed when moving from a three-profile solution to a four-profile solution at both time points. The BLRT test showed that the more profiles, the better the solutions, yet the five-profile solution started to have profiles representing less than 5% of the participants (Nylund et al. 2007). Thus, the four-profile solution was chosen as the final solution at each time point when fit indices, the interpretations of the profiles, and the added value of an extra profile were considered together. Next, the four-profile solution was compared between two grades (see Table 2). The partial distributional similarity was retained by setting the intercept of the first item of grit (i.e., one item of grit-consistency) to be equal across grades. This partial distributional similarity model had the lowest CAIC and BIC, implying that the four profiles in each grade were similar in terms of number, mean, variance, and group size.

**Table 2:** Results from latent profile analyses and latent transition analyses

Model	LL	#fp	Scaling	AIC	CAIC	BIC	ABIC	Entropy
*Final Latent Profile Analyses*								
8th grade	−1859.832	27	1.1093	3773.664	3916.983	3889.982	3804.273	0.802
9th grade	−1991.451	27	1.1665	4036.901	4180.221	4153.22	4067.511	0.825
*Longitudinal Latent Profile Analyses*								
Configural Similarity	−3851.283	54	1.1379	7810.565	8097.203	8043.202	7871.784	0.813
Structural Similarity	−3907.604	42	1.4163	7899.207	8122.148	8080.147	7946.822	0.783
Partial Structural Similarity	−3864.899	47	1.1071	7823.798	8073.279	8026.279	7877.081	0.817
Partial Dispersion Similarity	−3882.361	35	1.3481	7834.721	8020.505	7985.505	7874.4	0.788
Partial Distributional Similarity	−3887.44	32	1.349	7838.88	8008.739	7976.739	7875.158	0.788
*Latent Transition Analysis*	−1111.393	15	0.800	2252.785	2332.407	2317.407	2269.790	0.732
*Predictive Similarity* (Education Aspiration)								
Profile-specific Free Relations with Predictor	−574.265	33	0.5902	1214.529	1369.755	1336.754	1232.098	0.761
Free Relations with Predictor	−581.676	21	0.8693	1205.351	1304.131	1283.131	1216.531	0.749
Equal Relations with Predictor	−583.896	18	0.8074	1203.793	1288.460	1270.461	1213.375	0.746
*Explanatory Similarity*								
Free Relations with Outcome (without covariates)	−2334.559	25	0.9972	4719.118	4851.82	4826.821	4747.46	0.752
Equal Relations with Outcome (without covariates)	−2339.456	21	1.0907	4720.912	4832.382	4811.382	4744.719	0.751
Free Relations with Outcome (with covariates)	−1473.09	34	0.9914	3014.18	3187.438	3153.438	3045.537	0.756
Equal Relations with Outcome (with covariates)	−1479.995	30	0.9836	3019.991	3172.865	3142.865	3047.658	0.753

*LL* model loglikelihood; *#fp* number of free parameters; *scaling* scaling correction factor associated with robust maximum likelihood estimates; *AIC* Akaïke information criteria; *CAIC* constant AIC; *BIC* Bayesian information criteria; *ABIC* sample size adjusted BIC

**Fig. 1:**
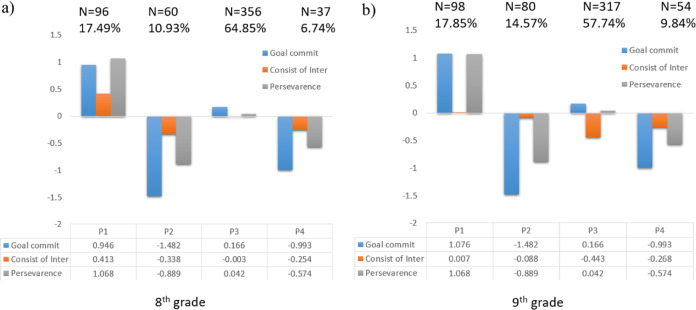
Profiles of academic-goal commitment and grit in 8th grade and 9th grade based on partial distributional similarity model. P1 = High committed-persistent and moderate consistency; P2 = Extremely low committed-persistent and moderate-low consistency; P3 = Moderate; P4 = Low committed-persistent and moderate-low consistency

This final model of partial distributional similarity is illustrated in Fig. 1 and was retained for interpretation and for the next stages of analyzes. Profile 1 presents high levels of academic goal commitment and grit-perseverance, and a moderate level of grit-consistency. This profile was labeled *High committed-persistent and moderate consistency* and characterized 17.49 and 17.85% of the participants in the eighth and ninth grades, respectively. Profile 2 displayed very low levels of commitment and grit-perseverance, and moderately low levels of grit-consistency. This *Extremely low committed-persistent and moderate-low consistency* profile characterized 10.93% of the eighth graders and 14.57% of the ninth graders. Profile 3 presented the average levels of all the indicators (except the moderately low level of grit-consistency in the ninth grade). This profile was labeled *Moderate* and characterized 64.85 and 57.74% of the eighth and ninth graders, respectively. Finally, Profile 4 was characterized by low levels of commitment and grit-perseverance and moderately low levels of grit-consistency. This *Low committed-persistent and moderate-low consistency* profile represented 6.74 and 9.84% of students in the eighth and ninth grades, respectively.

### Latent Transitions between Profiles

**Fig. 2:**
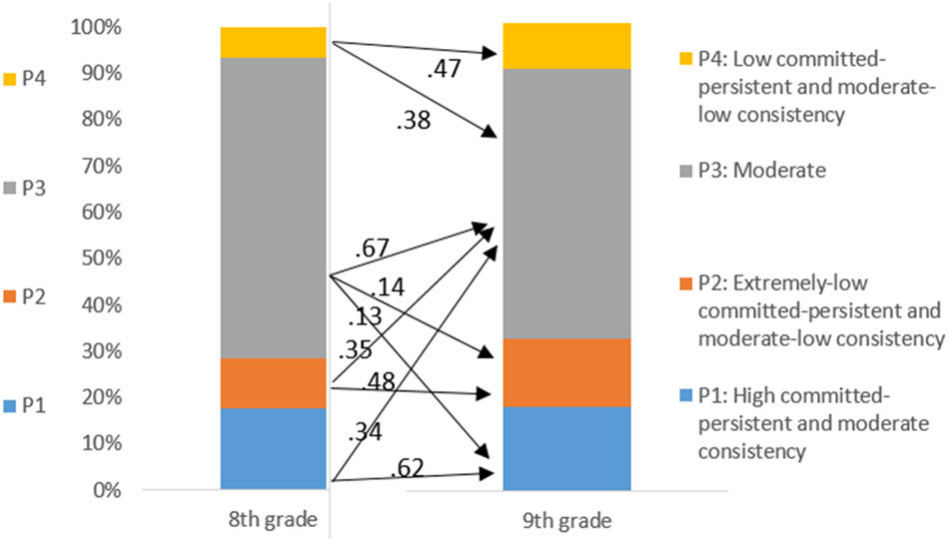
Transition probabilities from 8th grade to 9th grade

The final model of partial distributional similarity was then converted into an LTA using the manual auxiliary three-step approach (Asparouhov and Muthén 2014; Morin and Litalien 2017; Nylund-Gibson et al. 2014). The transition probabilities from this LTA are reported in Table 3 and Fig. 2. These results show that the High committed-persistent and moderate consistency profile was relatively stable (stability of 62.3%) and rarely became the Extremely low committed-persistent and moderate-low consistency profile (3.3%) or the Low committed-persistent and moderate-low consistency (0%) profile one year later. There was a 34.4% possibility that the High committed-persistent and moderate consistency profile would change into the Moderate profile. Regarding the Extremely low committed-persistent and moderate-low consistency profile, 47.7% remained in this profile, 35.1% shifted to the Moderate profile, and 17.2% shifted to the Low committed-persistent and moderate-low consistency profile one year later. No one switched from the Extremely low committed-persistent and moderate-low consistency profile to the High committed-persistent and moderate consistency profile.

**Table 3:** Transition probabilities for final latent transition analyses

	Transitions probabilities to *9th* *grade* profiles			
	High committed-persistent and moderate consistency	Extremely low committed-persistent and moderate-low consistency	Moderate	Low committed-persistent and moderate-low consistency
*8th* *grade*				
High committed-persistent and moderate consistency	0.623	0.033	0.344	0.00
Extremely low committed-persistent and moderate-low consistency	0.00	0.477	0.351	0.172
Moderate	0.13	0.139	0.671	0.059
Low committed-persistent and moderate-low consistency	0.00	0.151	0.384	0.465

The Moderate profile was also relatively stable (67.1%): 13% shifted to the High committed-persistent and moderate consistency profile, only 13.9% to the Extremely low committed-persistent and moderate-low consistency profile, and 5.9% to the Low committed-persistent and moderate-low consistency profile. Finally, the Low committed-persistent and moderate-low consistency profile was not very stable (46.5%): 38.4% changed to the Moderate profile and 15.1% to the Extremely low committed-persistent and moderate-low consistency profile. No one changed from the Low committed-persistent and moderate-low consistency profile to the High committed-persistent and moderate consistency profile.

### Predictors of Goal Commitment-Grit Profiles

To examine the predictors of profile membership and to test whether the results were consistent across the two grades, predictive similarity models were run on the basis of the partial distributional LTA model. As shown in Table 2, the predictive similarity model was attained on the basis of information criteria. In Table 4, multinomial logistic regression estimations show the effects of educational aspiration, gender and SES on the profiles.

**Table 4:** Results from multinomial logistic regressions for effects of predictors and demographics on profile memberships

	β (SE)	OR	β (SE)	OR	β (SE)	OR
	High committed-persistent and moderate consistency *vs*. Low committed-persistent and moderate-low consistency		Extremely low committed-persistent and moderate-low consistency *vs*. Low committed-persistent and moderate-low consistency		Moderate *vs*. Low committed-persistent and moderate-low consistency	
Education Aspiration	0.48 (0.29)	1.62	−0.45 (0.26)+	0.636*	0.39 (0.25)	1.469
Gender	−0.72 (0.31)*	0.485**	−0.16 (0.31)	0.849	−0.582 (0.266)*	0.559**
SES	0.442 (0.209)*	1.555+	−0.405 (0.172)*	0.667**	0.03 (0.15)	1.031
	High committed-persistent and moderate consistency *vs*. Moderate		Extremely low committed-persistent and moderate-low consistency *vs*. Moderate		High committed-persistent and moderate consistency vs. Extremely low committed-persistent and moderate-low consistency	
Education Aspiration	0.10 (0.24)	1.099	−0.838 (0.224)**	0.433**	0.932 (0.27)**	2.54*
Gender	−0.143 (0.216)	0.867	0.417 (0.228)+	1.518	−0.56 (0.27)*	0.57**
SES	0.411(0.16)*	1.509*	−0.435 (0.129)**	0.647**	0.847 (0.194)**	2.331**

*SE* standard error of the coefficient; *OR* odds ratio; *Gender* 1 = female, 2 = male

*p < 0.05; **p < 0.01

The results show that the adolescents in the Extremely low committed-persistent and moderate-low consistency profile were less likely to hold high educational aspirations than those in the Low committed-persistent and moderate-low consistency profile (OR = 0.64) and the Moderate profile (OR = 0.43). Adolescents from the High committed-persistent and moderate consistency profile group were 2.54 times more likely to have high educational aspirations than those from the Extremely low committed-persistent and moderate-low consistency profile. In addition, boys were less likely than girls to be in the High committed-persistent and moderate consistency profile (OR = 0.49) or Moderate profile (OR = 0.56) than in the Low committed-persistent and moderate-low consistency profile. Again, boys were less likely than girls to be in the High committed-persistent and moderate consistency profile group (OR = 0.57) than in the Extremely low committed-persistent and moderate-low consistency profile. Finally, adolescents from high SES families were 1.5 to 2.3 times more likely to be in the High committed-persistent and moderate consistency profile than in any other profile. In contrast, high SES adolescents were less likely to be in the Extremely low committed-persistent and moderate-low consistency profile than in the Moderate profile (OR = 0.65) or Low committed-persistent and moderate-low consistency profile (OR = 0.67). The differentiated role of educational aspirations, gender and SES in profile memberships further supports our four-profile solution.

### Goal Commitment-Grit profiles and Academic Achievement

To test the differences between the profiles in terms of academic achievement (i.e., GPA), explanatory similarity models were run on the basis of the partial distributional LTA model. To examine the similarity between the explanatory models in the eighth and ninth grade, two models were compared; one where outcomes were estimated freely across time points and one where outcomes were constrained as equal. Then, two sets of explanatory similarity models were tested; one that did not control for covariates (i.e., gender, SES, conscientiousness, academic persistence) and one that did. As shown in Table 2, among the models without covariates, the explanatory similarity model had the lowest values in all information criteria of all the alternative models, indicating that the mean differences between the profiles can be generalized from the eighth grade to the ninth grade. Students in the High committed-persistent and moderate consistency profile had the highest GPA, which was significantly higher than GPAs from students in the Extremely low committed-persistent and moderate-low consistency and the Low committed-persistent and moderate-low consistency profile groups (Cohen’s d = 1.144 and 1.023, respectively; see Table 5). Once the covariates were added, the explanatory similarity model was also supported (see Table 2). The results further disclosed that students from the High committed-persistent and moderate consistency profile still had the highest GPA and this GPA remained significantly higher than those from other profiles (see Table 5). Thus, the results suggested that a high level of both academic goal commitment and grit-PE as well as a moderate level of grit-CI produced the highest GPA. These results were consistent across the two grades.

**Table 5:** Goal commitment-grit profiles and academic achievements

	P1: High committed-persistent and moderate consistency M [CI]	P2: Extremely low committed-persistent and moderate-low consistency M [CI]	P3: Moderate M [CI]	P4: Low committed-persistent and moderate-low consistency M [CI]	Significant test
*Without covariates*					
**GPA**	8.748 [8.592; 8.905]	7.763 [7.319; 8.207]	8.556 [8.397; 8.716]	7.986 [7.75; 8.221]	P1 = P3 > P2 = P4
					Cohen’s d_p1-p2_ = 1.144
					Cohen’s d_p1-p3_ = 0.27
					Cohen’s d_p1-p4_ = 1.023
					Cohen’s d_p2-p3_ = 0.921
					Cohen’s d_p2-p4_ = 0.238
					Cohen’s d_p3-p4_ = 0.768
*With covariates*^a^					
**GPA**	8.574 [8.27; 8.83]	8.17 [7.843; 8.444]	8.442 [8.145; 8.691]	8.126 [7.803; 8.398]	P1 > P3 > P2 = P4
					Cohen’s d_p1-p2_ = 0.505
					Cohen’s d_p1-p3_ = 0.151
					Cohen’s d_p1-p4_ = 0.580
					Cohen’s d_p2-p3_ = 0.357
					Cohen’s d_p2-p4_ = 0.061
					Cohen’s d_p3-p4_ = 0.429

*M* mean; *CI* 95% confidence interval

^a^Covariates are Gender, SES, Conscientiousness, and Academic Persistence

### Results Robustness Check

When using complete two-wave data which consists of both grit and goal commitment (*N* = 549) for this study, the samples were reduced from 1171 in the eighth grade and from 765 in the ninth grade. Thus, a results robustness check was conducted to see whether the core findings could be replicated when using the original data points. During the first step, the factor scores of grit and goal commitment were saved from the longitudinal measurement invariance model (See Supplementary Table S3). As Mplus imputed the missing data automatically when saving the factor scores, the imputed data points were manually deleted to keep the original data size. Next, a series of LPAs were performed to determine the best profile solution for the eighth and ninth grades separately. As a third step, longitudinal latent profile analyses and latent transition analyses were tested. However, Mplus again imputed the missing data automatically, which lead to 1388 data points for each time wave. At this stage, deleting the imputed data points would not have solved the problem, as the imputed data as a whole resulted in the information criterion determining the quality of the model. In other words, if the longitudinal LPA models had too much imputed data (1171->1388 for the eighth grade, 765 -> 1388 for the ninth grade), the information criteria (e.g., BIC, aBIC) of these models may not be trustworthy. Therefore, no longitudinal LPA or LTA results can be reported here. Since no similar LPA models could be developed, the explanatory models were tested separately for the eighth and ninth grades.

In general, all information criteria continued to improve as the profile number increased (See Supplementary Table S4 and Supplementary Fig. S2). For the eighth grade, the information criteria tended to change less (i.e., the elbow plot tended to flatten) after the six-profile solution. However, as the six-profile solution resulted in a group with extreme indicator values and a very small sample size (*N* = 13), a five-profile solution was finally chosen. Similar situations emerged in the ninth grade, resulting in the decision to use a five-profile solution for the ninth grade. The final latent profiles of the eighth and ninth grades are presented in Supplementary Fig. S3. In comparison to the above-reported four profiles, the original *eighth grade* data resulted in a similar four profiles as well as an additional profile that had an extremely high level of grit-perseverance. The five profiles resulting from the original *ninth grade* data were generally parallel to the reduced-data profiles. The study also found one additional profile with an extremely high level of grit-perseverance. Key findings were replicated when the profiles were compared in terms of academic achievement, and the profiles with high grit-perseverance and academic goal commitment had the highest academic achievement. In fact, this profile was much better at achievement than the profiles with low grit-perseverance and academic goal commitment (Cohen’s d ranged from 0.502 to 1.082; See Supplementary Tables S5 and S6). These results held after controlling for gender, SES, conscientiousness, and academic persistence.

## Discussion

Although extant literature has reported a weak association between grit and achievement, most studies have overlooked two of the important components of grit: the goal and an individual’s corresponding level of commitment. This study intended to measure grit in tandem with goal commitment to show their association with achievement. The results showed that the adolescents in the groups with high commitment to academic-related goals, high grit-perseverance, and moderate grit-consistency yielded the highest levels of academic achievement. The study also indicated that grit-goal profiles were moderately stable, but affected by gender, SES, and educational aspirations. Overall, this study extends the existing literature on grit by measuring it alongside goal commitment, creating grit-goal profiles, and examining the transition, antecedents, and predictive utility of these profiles.

### Profiles of Grit and Goal Commitment and Achievement

Through person-oriented analyses, the present study found four profiles of grit and goal commitment across two grades. However, the “best” profile in terms of achievement, High committed-persistent and moderate consistency profile— represented less than 20% of participating adolescents. This study is one of the first to present information on the relation between grit and goal commitment. Since the combination of high grit and high goal commitment appears to be rare, this study offers some critical explanations as to why many other studies have found only weak associations between grit and achievement.

In this study, goal commitment and grit-perseverance varied together, whereas grit-consistency functioned more independently. Grit-consistency was mostly at a moderate level, while grit-perseverance and goal commitment were dynamic, thus reflecting variegated psychometric properties (Guo et al. 2019) and predictive power (Credé et al. 2017; Tang et al. 2019). The fact that grit-consistency was not tightly coupled with grit-perseverance may explain why researchers have had such difficulty using grit to predict achievement outcomes. Our findings corroborate the ongoing call for further examination of and attention to grit-consistency in regard to its conceptualization and measurement (Credé 2018).

### Shifting of Profiles in Critical Pre-Transition Year

In addition to presenting the grit and goal commitment profiles, this study furthers the understanding of shifts in these profiles from a developmental perspective. Although the time elapsed between measures was only one year, intensive changes were observed. The High committed-persistent and moderate consistency profile showed moderate stability (*prob* = 0.62) and was likely (*prob* = 0.34) to transition into the Moderate profile. The Moderate profile was stable (*prob* = 0.67) but it could become other profiles either as “best” or “worst” in terms of achievement (*prob* = 0.13–0.14). The other two profiles shifted to the Moderate profile one year later at a rate of about 35%. Though changes in profiles are to be expected, these findings reveal the likelihood of developmental shifts in grit-goal profiles as well as information on the mechanisms through which shifts occurred (i.e., for whom did profiles change and in which direction). This knowledge can be used to design interventions that better target specific groups of adolescents.

Although a single year of data does not permit extrapolation of our results to the full developmental period of adolescence, it is important to note that the eighth and ninth grades are critical pre-transition period for Finnish adolescents’ development in terms of goal setting and the pursuit of future educational and occupational success (OECD 2015; Salmela-Aro 2009). With ninth grade marking the end of compulsory education in Finland, our adolescent participants were approaching a point in their lives when they will have to choose whether to end their education or embark on an academic, vocational, or combined high-school track. In Finland, only ~50% of students enter the academic track (Tuominen-Soini et al. 2012; Tynkkynen et al. 2012), and in the year preceding this transition, some students begin to lose their commitment to their academic goals while others start to reaffirm their pursuit of an academic goal. The observed intensive changes in profiles before the transition, together with the low likelihood of the high committed-persistent profile, may further explain why it is hard to find strong associations between grit and academic achievement in adolescence. Although this study covers eighth and ninth graders from Finland, the findings can be extended to adolescents in other contexts during the transition phases. For instance, it is foreseeable that high school students from the US may in the similar situations where they’re experiencing normative developmental declines in achievement goals in conjunction with increasingly stressful, consequential decisions for their future.

### Antecedents of Profiles

In addition to creating goal-grit profiles, establishing their associations with achievement, and identifying how these profiles shift over a one-year period, this study examined the antecedents of the profiles. It was found that educational aspirations, gender, and SES are all factors that can distinguish profile memberships. Adolescents who were members of the high committed-persistent and moderate consistency group were most likely to be female, have high educational aspirations, and come from a high SES family background. Studies have shown that educational aspirations and SES are positively associated with academic-related goals (Massey et al. 2008) and grit (Usher et al. 2019; Verdesco 2016); thus, it was not surprising to find these patterns in our results as well. In general, there were no gender differences in grit (Credé et al. 2017), although girls were more likely to show high commitment to academic-related goals (Massey et al. 2008). This heightened commitment to academic goals may explain why girls were more highly represented in the High committed-persistent and moderate consistency profile than in the other grit-goal profiles. These findings not only indicate the optimal profile for student achievement, but it also implies that boys from low SES families may stand to benefit most from interventions addressing grit, goal setting, goal commitment, and achievement.

### Implications

The study findings have several important implications for policy, practice, and future research. Researchers, policy makers, and practitioners who aim to help adolescents achieve success at school should consider addressing academic-related goal commitment and grit simultaneously. In addition, it is critical for stakeholders to recognize the challenge adolescents face in balancing strong commitment to an academic-related goal with a high level of grit, as our findings indicate that most adolescents will experience declines in at least one of these characteristics over the course of adolescence. By acknowledging that it is difficult to simultaneously maintain both high commitment and high grit, stakeholders should make constant efforts to help adolescents develop grit, form and hold goals. This study also implies that, despite the difficulties, fostering adolescents’ educational aspirations is a possible way in which to help them form a high level of academic goal commitment and grit. Finally, if high goal commitment and high grit are both necessary to achieve in any given domain, then developing domain-specific grit scales so as to better assess participants’ goals, commitment, and grit is an important future step for grit researchers.

### Limitations

Several limitations need to be considered when interpreting this study’s results. First, the study focused on the commitment of academic-related goals; yet, these goals were self-defined by the participants and were thus qualitatively different from each other. It is foreseeable that a goal of “I want to get an A+ in physics” means something inherently different than “I want to improve my math grade to a B+”. Such nuanced language may consequently affect goal commitment. Unfortunately, goal contents were not examined in this study due to limited resources. However, our findings did show that having a committed goal (in comparison to not having one) made a difference in achievement when coupled with grit. Second, the study used the complete sample (*N* = 549) across two grades instead of the original and lager sample in each grade (*N* = 1171 and *N* = 765, respectively for 8th and 9th grade) due to concerns about missing data procedures for mixture modeling (Cetin-Berber and Leite 2018; Sterba 2016). The high attrition rate may have reduced statistical power when using the “smaller” complete sample. Third, given that one year is too short to be able to fully understand grit and goal commitment from a developmental perspective, future longitudinal studies are still needed to examine these relationships, especially those that span multiple transition periods.

## Conclusion

By addressing the weak grit-achievement association and noting the lack of goal components in grit scales and previous studies, the current study provides crucial findings for the future study and understanding of grit’s complicated relation with achievement. Using a person-oriented approach, this study found that gritty adolescent students who were also highly committed to an academic goal had higher academic achievements. Notably, only a small fraction of students (<20%) simultaneously had high commitment to an academic goal and high grit. In addition, this group of students was modestly stable that about one third of them dropped their commitment to academic learning or the level of grit before they moved into upper secondary schools. Thus, the study offers some critical explanations on the weak grit effect and indicates that modifying the grit scale may a viable way to observe strong grit effect.

## Supplementary information

Supplementary Materials

## Footnotes

1 10 is the highest grade in the Finnish grading system

2 More variables/data (e.g., engagement and burnout) was deposited in the OSF; they were kept due to examinations for other research outputs.
