# Supplementary material for: Putting the Goal Back into Grit: Academic Goal Commitment, Grit, and Academic Achievement
Source: J Youth Adolesc. 2020 Nov 17;50(3):470–84. doi: 10.1007/s10964-020-01348-1 (PMC7910368; doi:10.1007/s10964-020-01348-1)
Supplement: Supplementary file 1 — Supplementary Materials [file 10964_2020_1348_MOESM1_ESM.docx]

**Online Supplemental Materials for:**

**Putting the Goal Back into Grit: Academic Goal Commitment, Grit and Academic Achievement**

**Contents**

[**Table S1** *Goodness-of-Fit Statistics of the Preliminary Measurement Models* 2](#_Toc49420112)

[**Table S2** *Results from the Latent Profile Analysis Models Estimated Separately at Each Time Point* 3](#_Toc49420113)

[**Figure S1** *Elbow Plot of the Value of the Information Criteria for Solutions Including Different Number of Latent Profiles (Grade 8)* 4](#_Toc49420114)

[**Figure S2** *Elbow Plot of the Value of the Information Criteria for Solutions Including Different Number of Latent Profiles (Grade 9)* 4](#_Toc49420115)

[**Table S3** *Goodness-of-Fit Statistics of the Preliminary Measurement Models for Original data* 5](#_Toc49420116)

[**Table S4** *Results from the Latent Profile Analysis Models Estimated Using Original Data* 6](#_Toc49420117)

[**Figure S2a & S2b** *Elbow Plot of the Value of the Information Criteria for Solutions Including Different Number of Latent Profiles from Original Data* 7](#_Toc49420118)

[**Figure S3a & 3b** *Profiles of Academic-goal and Grit in 8^th^ grade and 9^th^ grade from original data* 8](#_Toc49420119)

[**Table S5** *Goal commitment-grit profiles and academic achievements for 8^th^ grade from original data* 9](#_Toc49420120)

[**Table S6** *Goal commitment-grit profiles and academic achievements for 9^th^ grade from original data* 10](#_Toc49420121)

**Table S1** *Goodness-of-Fit Statistics of the Preliminary Measurement Models*

| Description | *χ*² (*df*) | CFI | TLI | RMSEA | 90% CI | MD *∆χ²* (*df*) | ∆CFI | ∆TLI | ∆RMSEA |
| --- | --- | --- | --- | --- | --- | --- | --- | --- | --- |
| *Measurement Models* |  |  |  |  |  |  |  |  |  |
| Grade 8 (N = 549) | 191.343(41) | .883 | .843 | .082 | [.07; .094] | - | - | - | - |
| Grade 9 (N = 549) | 153.743(41) | .921 | .894 | .071 | [.059; .083] | - | - | - | - |
| Longitudinal configural invariance (N = 549) | 452.189 (183) | .92 | .899 | .052 | [.046; .058] | - | - | - | - |
| Longitudinal weak invariance | 465.979 (191) | .918 | .901 | .051 | [.045; .057] | 14.106 (8) | -.002 | .002 | -.001 |
| Longitudinal strong invariance | 516.722 (199) | .906 | .89 | .054 | [.048; .06] | 54.882 (8)* | -.012 | -.011 | .003 |
| Longitudinal partial strong invariance (free intercept of grit1) | 503.129 (198) | .909 | .894 | .053 | [.047; .059] | 39.884 (7)* | -.009 | -.007 | .002 |

*Note.* * *p*< .01; *χ*²: chi-square test of exact fit; *df*: degrees of freedom; CFI: comparative fit index; TLI: Tucker-Lewis index; RMSEA: root mean square error of approximation; 90% CI: 90% confidence interval; MD *∆χ*²: chi-square difference tests calculated with Mplus’ DIFFTEST function.

**Table S2** *Results from the Latent Profile Analysis Models Estimated Separately at Each Time Point*

| Model | LL | #fp | Scaling | AIC | CAIC | BIC | ABIC | Entropy | aLMR | BLRT |
| --- | --- | --- | --- | --- | --- | --- | --- | --- | --- | --- |
| Time 1 (N = 549) | |  |  |  |  |  |  |  |  |  |
| 1 Profile | -2139.375 | 6 | 1.2746 | 4290.75 | 4322.599 | 4316.598 | 4297.552 | Na | Na | Na |
| 2 Profiles | -1996.993 | 13 | 1.3326 | 4019.986 | 4088.991 | 4075.992 | 4034.724 | .607 | .0032 | < .001 |
| 3 Profiles | -1922.062 | 20 | 1.148 | 3884.123 | 3990.286 | 3970.285 | 3906.797 | .698 | .0019 | < .001 |
| 4 Profiles | -1859.832 | 27 | 1.1093 | 3773.664 | 3916.983 | 3889.982 | 3804.273 | .802 | .0117 | < .001 |
| 5 Profiles | -1813.083 | 34 | 1.1061 | 3694.166 | 3874.641 | 3840.641 | 3732.711 | .815 | .0354 | < .001 |
| Time 2 (N = 549) | |  |  |  |  |  |  |  |  |  |
| 1 Profile | -2350.959 | 6 | 1.1905 | 4713.917 | 4745.767 | 4739.766 | 4720.719 | Na | Na | Na |
| 2 Profiles | -2203.827 | 13 | 1.4531 | 4433.654 | 4502.659 | 4489.659 | 4448.392 | .654 | .0179 | < .001 |
| 3 Profiles | -2099.803 | 20 | 1.2524 | 4239.605 | 4345.768 | 4325.767 | 4262.279 | .77 | .0023 | < .001 |
| 4 Profiles | -1991.451 | 27 | 1.1665 | 4036.901 | 4180.221 | 4153.22 | 4067.511 | .825 | < .001 | < .001 |
| 5 Profiles | -1950.654 | 34 | 1.216 | 3969.308 | 4149.783 | 4115.783 | 4007.853 | .782 | .0882 | < .001 |

Note. LL: model loglikelihood; #fp: number of free parameters; scaling: scaling correction factor associated with robust maximum likelihood estimates; AIC: Akaïke information criteria; CAIC: constant AIC; BIC: Bayesian information criteria; ABIC: sample size adjusted BIC; aLMR: adjusted Lo-Mendel-Rubin likelihood ratio test; BLRT: bootstrap likelihood ratio test.

# **Figure S1** *Elbow Plot of the Value of the Information Criteria for Solutions Including Different Number of Latent Profiles (Grade 8)*

# **Figure S2** *Elbow Plot of the Value of the Information Criteria for Solutions Including Different Number of Latent Profiles (Grade 9)*

**Table S3** *Goodness-of-Fit Statistics of the Preliminary Measurement Models for Original data*

| Description | *χ*² (*df*) | CFI | TLI | RMSEA | 90% CI | MD *∆χ²* (*df*) | ∆CFI | ∆TLI | ∆RMSEA |
| --- | --- | --- | --- | --- | --- | --- | --- | --- | --- |
| *Measurement Models* |  |  |  |  |  |  |  |  |  |
| Grade 8 (N = 1171) | 262.358(41) | .926 | .900 | .068 | [.06; .076] | - | - | - | - |
| Grade 9 (N = 765) | 168.528(41) | .933 | .910 | .064 | [.054; .074] | - | - | - | - |
| Longitudinal configural invariance | 541.451(183) | .937 | .921 | .038 | [.034; .041] | - | - | - | - |
| Longitudinal weak invariance | 562.212(191) | .935 | .921 | .037 | [.034; .041] | 21.035(8)* | -0.002 | 0 | -0.001 |
| Longitudinal strong invariance | 660.838(199) | .919 | .906 | .041 | [.037; .044] | 109.379(8)* | -0.016 | -0.015 | 0.004 |
| Longitudinal partial strong invariance (free intercept of grit1) | 624.233(198) | .925 | .913 | .039 | [.036; .043] | 67.933(7)* | -0.01 | -0.008 | 0.002 |

*Note.* * *p*< .01; *χ*²: chi-square test of exact fit; *df*: degrees of freedom; CFI: comparative fit index; TLI: Tucker-Lewis index; RMSEA: root mean square error of approximation; 90% CI: 90% confidence interval; MD *∆χ*²: chi-square difference tests

**Table S4** *Results from the Latent Profile Analysis Models Estimated Using Original Data*

| Model | LL | #fp | Scaling | AIC | CAIC | BIC | ABIC | Entropy | aLMR | BLRT |
| --- | --- | --- | --- | --- | --- | --- | --- | --- | --- | --- |
| Time 1 (N = 1171) | |  |  |  |  |  |  |  |  |  |
| 1 Profile | -4423.27 | 6 | 1.1868 | 8858.539 | 8894.934 | 8888.933 | 8869.875 | #N/A | #N/A | #N/A |
| 2 Profiles | -4105.443 | 13 | 1.265 | 8236.885 | 8315.739 | 8302.738 | 8261.446 | 0.958 | 0 | 0 |
| 3 Profiles | -3812.892 | 20 | 1.2796 | 7665.783 | 7787.096 | 7767.096 | 7703.569 | 0.769 | 0.0021 | 0 |
| 4 Profiles | -3664.356 | 27 | 1.1171 | 7382.711 | 7546.484 | 7519.483 | 7433.721 | 0.837 | 0 | 0 |
| 5 Profiles | -3557.288 | 34 | 1.0423 | 7182.575 | 7388.807 | 7354.806 | 7246.81 | 0.806 | 0 | 0 |
| 6 Profiles | -3464.871 | 41 | 1.0514 | 7011.743 | 7260.432 | 7219.433 | 7089.203 | 0.803 | 0.0005 | 0 |
| 7 Profiles | -3427.584 | 48 | 1.0387 | 6951.168 | 7242.317 | 7194.318 | 7041.853 | 0.786 | 0.0007 | 0 |
| Time 2 (N = 765) | |  |  |  |  |  |  |  |  |  |
| 1 Profile | -3012.104 | 6 | 1.1798 | 6036.207 | 6070.047 | 6064.047 | 6044.994 | #N/A | #N/A | #N/A |
| 2 Profiles | -2816.579 | 13 | 1.3942 | 5659.157 | 5732.476 | 5719.476 | 5678.195 | 0.6 | 0.0037 | 0 |
| 3 Profiles | -2673.527 | 20 | 1.451 | 5387.053 | 5499.851 | 5479.851 | 5416.342 | 0.741 | 0.0683 | 0 |
| 4 Profiles | -2525.704 | 27 | 1.4765 | 5105.409 | 5257.685 | 5230.685 | 5144.948 | 0.822 | 0.0288 | 0 |
| 5 Profiles | -2457.324 | 34 | 1.2062 | 4982.648 | 5174.404 | 5140.404 | 5032.439 | 0.795 | 0.0461 | 0 |
| 6 Profiles | -2406.666 | 41 | 1.2578 | 4895.332 | 5126.567 | 5085.567 | 4955.374 | 0.812 | 0.0438 | 0 |
| 7 Profiles | -2353.57 | 48 | 1.3304 | 4803.14 | 5073.854 | 5025.854 | 4873.433 | 0.807 | 0.3634 | 0 |

Note. LL: model loglikelihood; #fp: number of free parameters; scaling: scaling correction factor associated with robust maximum likelihood estimates; AIC: Akaïke information criteria; CAIC: constant AIC; BIC: Bayesian information criteria; ABIC: sample size adjusted BIC; aLMR: adjusted Lo-Mendel-Rubin likelihood ratio test; BLRT: bootstrap likelihood ratio test.

# **Figure S2a & S2b** *Elbow Plot of the Value of the Information Criteria for Solutions Including Different Number of Latent Profiles from Original Data*

3a) 3b)


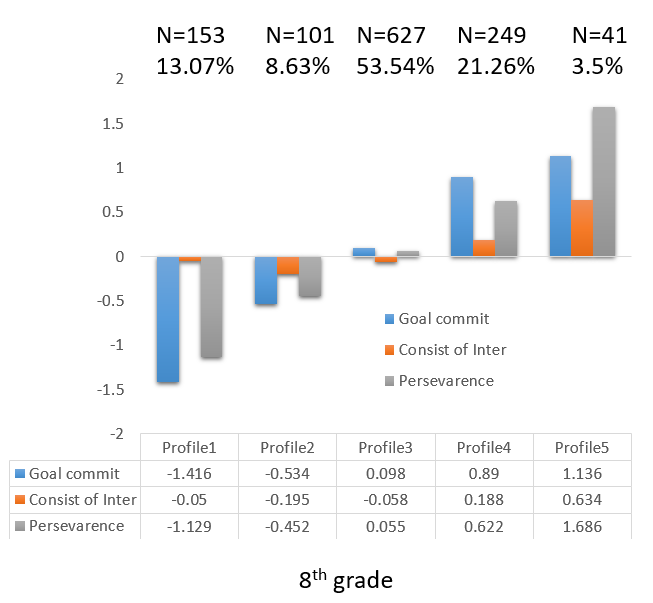

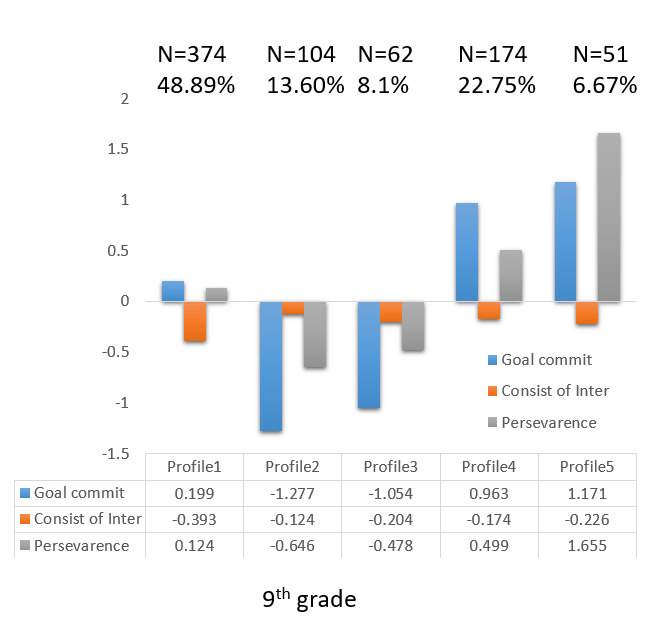


# **Figure S3a & 3b** *Profiles of Academic-goal commitment and Grit in 8^th^ grade and 9^th^ grade from original data*

**Table S5** *Goal commitment-grit profiles and academic achievements for 8^th^ grade from original data*

|  | Profile 1  M [CI] | Profile 2  M [CI] | Profile 3  M [CI] | Profile 4  M [CI] | Profile 5  M [CI] | Significant test |
| --- | --- | --- | --- | --- | --- | --- |
| *Without covariates* |  |  |  |  |  |  |
| GPA | 7.525  [7.312; 7.737] | 7.877  [7.695; 8.058] | 8.314  [8.225; 8.402] | 8.518  [8.391; 8.646] | 8.811  [8.562; 9.06] | P5>P4>P3>P2>P1  Cohen’s d_p1-p2_ = .356  Cohen’s d_p1-p3_ = .816  Cohen’s d_p1-p4_ = 1.137  Cohen’s d_p1-p5_ = 1.436  Cohen’s d_p2-p3_ = .423  Cohen’s d_p2-p4_ = .826  Cohen’s d_p2-p5_ = 1.147  Cohen’s d_p3-p4_ = .309  Cohen’s d_p3-p5_ = .629  Cohen’s d_p4-p5_ = .336 |
| *With covariates*^a^ |  |  |  |  |  |  |
| GPA | 7.484  [7.124; 7.843] | 7.899  [7.549; 8.249] | 8.096  [7.788; 8.403] | 8.177  [7.829; 8.525] | 8.398  [7.934; 8.861] | P5=P4; P5>P3=P2>P1; P4=P3;  Cohen’s d_p1-p2_ = .502  Cohen’s d_p1-p3_ = .749  Cohen’s d_p1-p4_ = .852  Cohen’s d_p1-p5_ = 1.082  Cohen’s d_p2-p3_ = .27  Cohen’s d_p2-p4_ = .38  Cohen’s d_p2-p5_ = .643  Cohen’s d_p3-p4_ = .108  Cohen’s d_p3-p5_ = .38  Cohen’s d_p4-p5_ = .278 |

Note. M: Mean; CI: 95% Conﬁdence Interval. Proﬁle 1: Extremely-low Committed; Proﬁle 2: Low Committed; Proﬁle 3: Moderate; Proﬁle 4: High Committed; Proﬁle 5: Extremely-high Committed-Persistent. ^a^Covariates are Gender, SES, Conscientiousness, and Academic Persistence

**Table S6** *Goal commitment-grit profiles and academic achievements for 9^th^ grade from original data*

|  | Profile 1  M [CI] | Profile 2  M [CI] | Profile 3  M [CI] | Profile 4  M [CI] | Profile 5  M [CI] | Significant test |
| --- | --- | --- | --- | --- | --- | --- |
| *Without covariates* |  |  |  |  |  |  |
| GPA | 8.551  [8.428; 8.674] | 8.002  [7.74; 8.264] | 7.912  [7.652; 8.172] | 8.615  [8.462; 8.767] | 8.705  [8.457; 8.953] | P5=P4=P1>P3=P2  Cohen’s d_p1-p2_ = .642  Cohen’s d_p1-p3_ = .753  Cohen’s d_p1-p4_ = .07  Cohen’s d_p1-p5_ = .171  Cohen’s d_p2-p3_ = .044  Cohen’s d_p2-p4_ = .717  Cohen’s d_p2-p5_ = .804  Cohen’s d_p3-p4_ = .841  Cohen’s d_p3-p5_ = .935  Cohen’s d_p4-p5_ = .105 |
| *With covariates*^a^ |  |  |  |  |  |  |
| GPA | 8.099  [7.7; 8.498] | 7.612  [7.122; 8.102] | 7.459  [6.972; 7.946] | 8.056  [7.642; 8.469] | 8.045  [7.558; 8.531] | P5=P4=P1>P3=P2  Cohen’s d_p1-p2_ = .658  Cohen’s d_p1-p3_ = .789  Cohen’s d_p1-p4_ = .112  Cohen’s d_p1-p5_ = .11  Cohen’s d_p2-p3_ = .06  Cohen’s d_p2-p4_ = .563  Cohen’s d_p2-p5_ = .573  Cohen’s d_p3-p4_ = .684  Cohen’s d_p3-p5_ = .698  Cohen’s d_p4-p5_ = .004 |

Note. M: Mean; CI: 95% Conﬁdence Interval. Proﬁle 1: Moderate; Proﬁle 2: Extremely-low Committed; Proﬁle 3: Low Committed; Proﬁle 4: High Committed; Proﬁle 5: Extremely-high Committed-Persistent. ^a^Covariates are Gender, SES, Conscientiousness, and Academic Persistence
